# Exploiting replication deficiencies to illuminate HIV: host interactions

**DOI:** 10.1186/1742-4690-8-S2-O6

**Published:** 2011-10-03

**Authors:** Michael Malim

**Affiliations:** 1 Kings College University, London, UK

## 

The replication of HIV-1 is controlled through multiple interactions with factors and pathways provided by infected host cells; many of these benefit the virus and promote effective replication, though others can function to inhibit specific steps of the life cycle. The seminar will describe recent work on one protein from each category. CRM1 is a nuclear export receptor that enables the HIV-1 Rev protein to transport unspliced viral RNA to the cytoplasm. Murine CRM1 is poorly active in the context of Rev function, and comparisons with the human counterpart have led to the identification of a novel aspect to Rev function which also bears the hallmark of an ancient host-pathogen interaction. APOBEC3G is a DNA cytidine deaminase that potently suppresses infection by HIV-1 as well as numerous other retro-elements. The presentation will summarise how this protein was assigned a role in HIV-1 replication, discuss recent studies on its mechanism of action, describe how HIV-1 evades this anti-viral factor through the action of its Vif protein, and speculate on how HIV-1 may even exploit APOBEC3G to its advantage under certain circumstances.

